# The Geography of Gender Inequality

**DOI:** 10.1371/journal.pone.0145778

**Published:** 2016-03-01

**Authors:** Brendan Fisher, Robin Naidoo

**Affiliations:** 1 Gund Institute, Rubenstein School of Environment and Natural Resources, Aiken Center, University of Vermont, Burlington, Vermont, United States of America; 2 World Wildlife Fund, Washington, DC, United States of America; 3 Institute for Resources, Environment and Sustainability, University of British Columbia, Vancouver, British Columbia, Canada; University of Modena & Reggio Emilia, ITALY

## Abstract

Reducing gender inequality is a major policy concern worldwide, and one of the Sustainable Development Goals. However, our understanding of the magnitude and spatial distribution of gender inequality results either from limited-scale case studies or from national-level statistics. Here, we produce the first high resolution map of gender inequality by analyzing over 689,000 households in 47 countries. Across these countries, we find that male-headed households have, on average, 13% more asset wealth and 303% more land for agriculture than do female-headed households. However, this aggregate global result masks a high degree of spatial heterogeneity, with bands of both high inequality and high equality apparent in countries and regions of the world. Further, areas where inequality is highest when measured by land ownership generally are not the same areas that have high inequality as measured by asset wealth. Our metrics of gender inequality in land and wealth are not strongly correlated with existing metrics of poverty, development, and income inequality, and therefore provide new information to increase the understanding of one critical dimension of poverty across the globe.

## Introduction

Reducing gender inequality is a major global policy concern. Goal 5 of the recently ratified Sustainable Development Goals is “*Achieve gender equality and empower all women and girls*.” The pathway under this goal (Goal 5a) is via reforms that give women equal rights and access to economic, financial and natural resources [1]. One reason gender equality is so high on the international policy agenda is the growing body of evidence showing that improving the welfare of women and closing the inequality gap can lead to improved childhood nutrition and reduced mortality [2–4], increased school enrollment [2,5], improved maternal and children’s health [4,6,7] and improved natural resource management [8–10]. Given this evidence accruing from around the world, promoting gender equality is viewed as a key strategy in development campaigns [11], to increase food security [12], reduce poverty [13], mitigate HIV and malaria transmission [14], and ensure sustainability [15].

In order to accelerate the impacts of policies and programs that relate to gender, a critical first step is to better understand how inequality is manifested around the world, in particular, by pinpointing where it occurs using approaches and indicators that are consistently disaggregated to as fine a resolution as possible. Unfortunately, such fine-scale indicators for gender inequality do not currently exist. And so while recent assessments of gender disparity suggest that in many parts of the world we have failed to deliver on Millennium Development Goal (MDG) III—“*promote gender equality and empower women*.” [16–18], with evidence suggesting that women in some countries have seen decreased opportunities to improve their welfare and that of their families (e.g. Vietnam: worsening wage equality; Pakistan: reduced political empowerment; ref.[19]), these indicators are inherently limited due to the coarseness of their resolution.

Here, we address this lack of fine-scale data by presenting high-resolution maps of gender inequality, using a consistent analytical approach that represents approximately half of the world’s least developed countries. For our measures of inequality we use land ownership and household wealth to compare welfare between male-headed and female-headed households across 30,509 sample clusters (which represent villages, communities or subsets of more populated areas, hereon *villages*). We chose these indicators since in many parts of the developing world, access to land is the critical input to a household’s welfare function, and asset wealth (e.g. housing materials, bicycle ownership, water source) reflects other forms of productive capital for the household. There are of course other measures of gender inequality reflected in national indexes (e.g. percentage of women in political leadership positions), but we focused on these two metrics since they provide a micro-view of the gender dynamics relevant to project or implementation scales, and because we can map and measure them with a consistent methodology.

## Methods

We used the Demographic and Health Surveys project [20] to assemble a dataset of household surveys from 30,509 sampling clusters encompassing over 689,000 households. DHS surveys are nationally-representative surveys of the health and wellbeing of a country's women, men and children. From this dataset we extracted two key metrics of a household’s welfare: first, the number of hectares owned that are available for agriculture; and next, a wealth asset index. Land is the most important production factor for households in much of the developing world [21], and so we assumed that all else being equal, households with more agricultural land are better off than those with less. The wealth index is an aggregate metric of the accumulation of physical capital related to human well-being per household; it includes a core set of assets measured across all countries (e.g., the status of housing, water supply, communications, and sanitation facilities), as well as country-specific indicators of welfare [22]. For both indices, we calculated a gender inequality ratio for each of the 30,509 villages/communities in our dataset by dividing the average land (wealth) value for households headed by males by the corresponding average value in those headed by females. Our data are not globally comprehensive, but because DHS surveys are designed to represent national populations in impoverished countries, they are a representative sample of over 70% of the people living in sub-Saharan Africa (the world's poorest region), as well as significant areas of Asia and Latin America.

## Results

**Fig 1:**
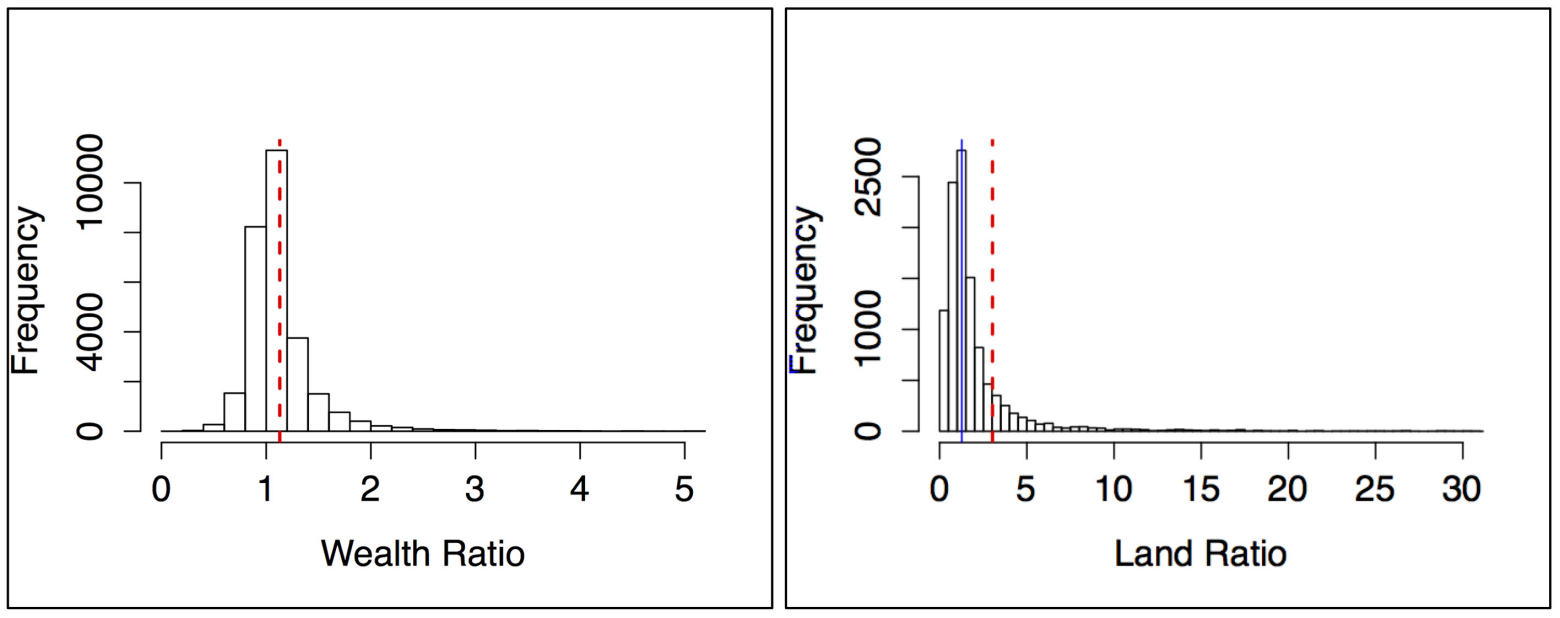
**Wealth and Land Inequality** A) Distribution of ratios of household asset wealth between male- and female-headed households at the village level [mean (red line) = 1.13, n = 30,509], B) Distribution of ratios of amount of land for cultivation between male- and female-headed households at the village level [mean (red dashed line) = 3.03, median (blue line) = 1.28, n = 11,809].

Our results show that, across the globe, households headed by males have on average 13% more asset wealth than their female counterparts, and on average own an astonishing 303% more land (Fig 1A and 1B). The land-ownership data is highly right-skewed, but even using the median, male-headed households have 28% more land wealth that female-headed ones. The extreme disparities in our results for land ownership demonstrate that a focus on common measurements of inequality (e.g. income) may greatly underestimate gender inequality when viewed through other dimensions of poverty.

At the national scale, male-headed households have significantly more asset wealth than their female counterparts in 17 of 47 countries (*t*-tests with *p* < 0.05), while only in Senegal did female-headed households have significantly more asset wealth than male-headed households (S1 Table in S1 File). For land ownership, male-headed households had significantly more land in 13 countries (*t*-tests with *p* < 0.05), while Senegal again was the only country where female-headed households owned significantly more land than those headed by males (S2 Table in S1 File). In the remaining 29 (for asset wealth) and 16 (for land ownership) countries, there were no statistically significant differences between male- and female-headed households.

**Fig 2:**
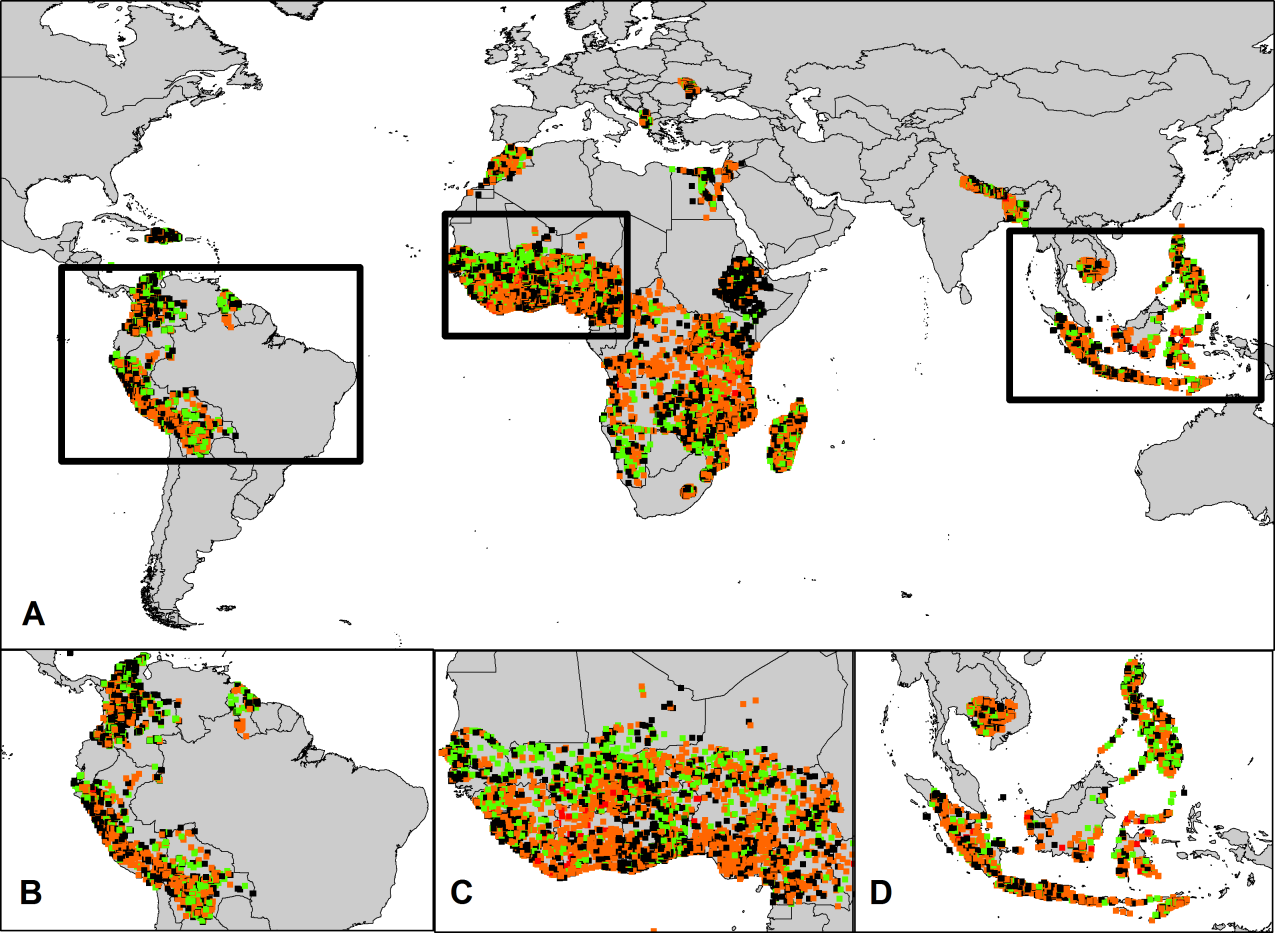
**Wealth inequality between male- and female-headed households** A: Global Results B: Latin America C: West Africa D: Southeast Asia. Green circles represent villages where female-headed households have significantly higher asset wealth. Black circles represent villages where there is no statistical difference in the asset wealth between male- and female-headed households. Orange and Red circles represent villages with high inequality, where female-headed households have significantly lower asset wealth. [Basemap Source: Country borders displayed from the World Borders Dataset under a Creative Commons Attribution-Share Alike License (http://thematicmapping.org/downloads/world_borders.php)]

**Fig 3:**
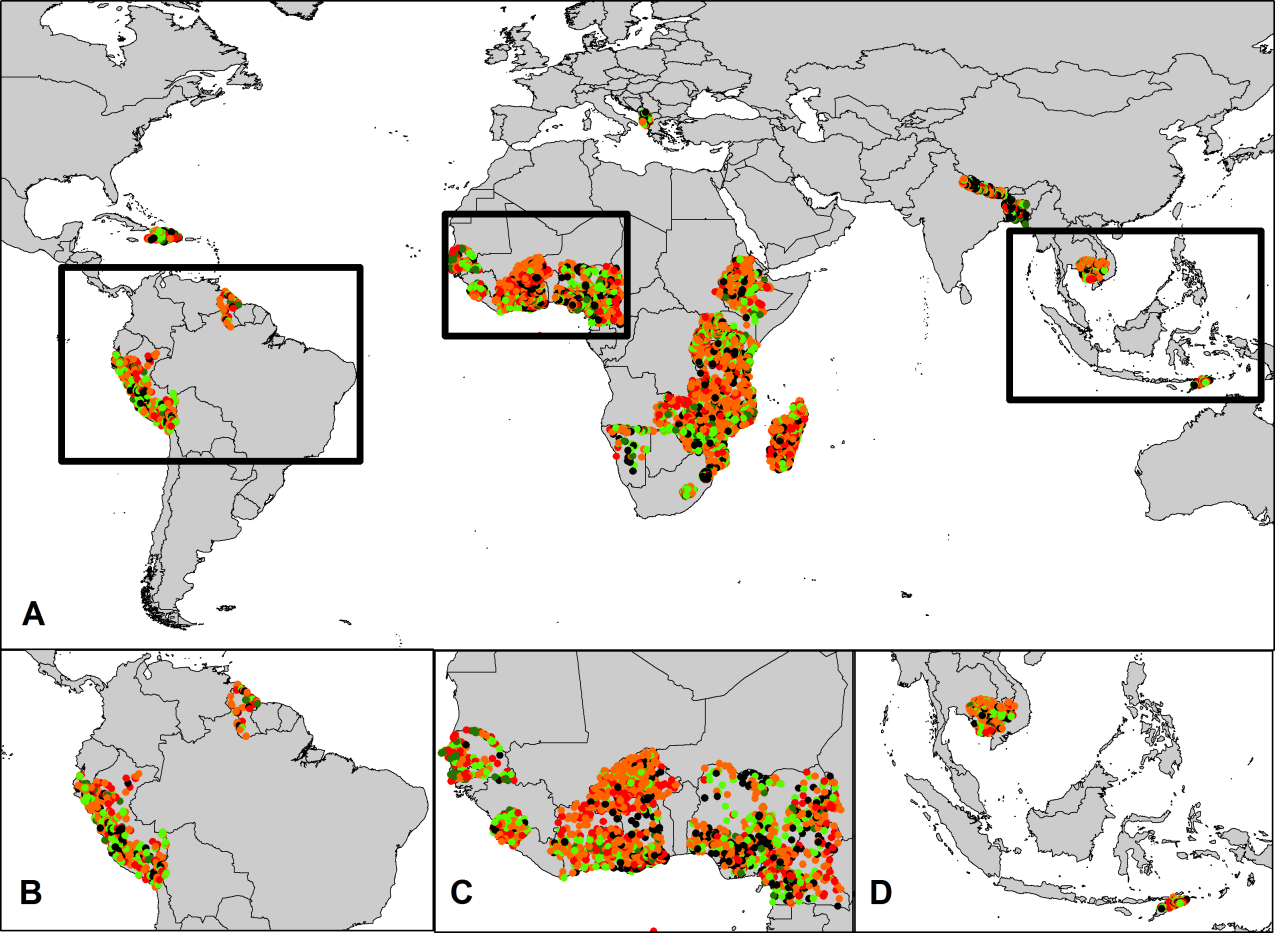
**Land inequality between male- and female-headed households** A: Global Results B: Latin America C: West Africa D: Southeast Asia. Green circles represent villages where female-headed households have significantly higher land wealth. Black circles represent villages where there is no statistical difference in the land wealth between male and female-headed households. Orange and Red circles represent villages with high inequality, where female-headed households have significantly less land. [Basemap Source: Country borders displayed from the World Borders Dataset under a Creative Commons Attribution-Share Alike License (http://thematicmapping.org/downloads/world_borders.php)]

Despite the significance of country-level differences in land and asset wealth between male and female-headed households, examining aggregate results at the national level masks the highly heterogeneous nature of gender inequality in huge swathes of the developing world. Both the land and wealth inequality ratios show strong spatial heterogeneity within, and across, countries (Figs 2A and 3A; [We zoom in on three areas for which we have results for multiple countries in a given area and where we further describe the results–Western South America, West Africa and Southeast Asia]). For example, while Senegal is the only country where female-headed households have significantly higher levels of both wealth and land, this national scale result is not consistent throughout the country. While female-headed households along the Senegal River (which forms Senegal’s northern border with Mauritania) do have higher wealth and greater access to land, male-headed households along the coast and in the capital, Dakar, are much wealthier (Fig 2C). And across the whole of West Africa, there exist belts of wealthier, female-headed households located 800–1000 km from the coast, while wealthier male-headed households are found 400–600 km away; both of these inequality belts extend from Senegal through several intervening countries all the way to Cameroon (S1 Fig in S1 File). Additional regional patterns are evident in South America, where there is a strong altitudinal gradient of inequality: households above 3000 m have wealth ratios significantly more skewed toward those headed by males than do households at lower elevations (S2 Fig in S1 File).

Such spatial heterogeneity contrasts with land and wealth ratios in other areas, such as in Cambodia, where large inequalities in both land ownership and wealth are consistent across the country (Figs 2D and 3D; S1 and S2 Tables in S1 File). Hence, Cambodia represents a case where averaged national level measurements of inequality might accurately reflect disaggregated, village scale results. Parts of east Africa, particularly Ethiopia and Kenya, also show spatially homogenous levels of inequality between male- and female-headed households. However, these countries are the exception rather than the rule.

In addition to the high degree of spatial variation in inequality within and across countries, there is also a lack of spatial concordance between our two inequality measures. For example, in both Peru and Tanzania, male-headed households have on average greater land ownership and higher asset wealth compared to female-headed households (S1 and S2 Tables in S1 File). Yet across all villages in both countries, the correlation between the two metrics is either poor or non-existent; villages where land inequality is high are not the same as those where asset inequality is high, and vice-versa (Peru: *r* = 0.058, *p* = 0.04, *n* = 1242; Tanzania: *r* = -0.003, *p* = 0.94, *n* = 380;) (Figs 2 and 3).

This lack of spatial correlation between land and asset wealth ratio holds across our entire dataset (*r* = -0.005, *p* = 0.62, *n* = 10,907). Given that poverty is multi-dimensional, the lack of association between our land and wealth metrics suggests that they represent different facets of poverty, and indicate the need for differential approaches for building livelihoods and resilience in extremely poor areas.

To test that our results did in fact represent differences related to the gender of household headship, rather than result from other potentially confounding factors, we conducted three additional analyses. First, we tested whether differences in male- versus female-headed households may instead have been an effect of rural versus urban residency, since in some countries males may migrate towards economic opportunities in urban areas, with females therefore more likely to head households in these countries' rural areas by default. This analysis included tests for the main effects of gender and of urban/rural residence, as well as an interaction term, and used both wealth and land ownership as response variables. After controlling for the fact that urban households are much wealthier and have more land ownership than rural households, the strong independent effect of gender was actually accentuated (S3 and S4 Tables in S1 File).

Second, we tested to see if our results might otherwise be explained by a difference in the number of people in the household. For example, if female households contained fewer dependents than male-headed households, we might reasonably conclude that land and wealth capital levels need to be scaled by household number. However, we found only slight differences in numbers of household inhabitants between male and female-headed households (i.e., a global average of 0.04 more inhabitants in male-headed households (S5 Table in S1 File)).

Third, looking at nine countries in Latin America, Deere et al. [23] found that household head analysis may underestimate the extent of female-owned wealth, since it does not account for asset and land ownership by females in households headed by males (e.g. Ecuador and Argentina). To test if our analysis based on headship does reflect gender inequality outside of households headed by women, we extracted information on decision-making in our dataset and compared responses on who makes key household decisions to the gender of the household head. We found that across all countries, males in male-headed households are significantly and consistently more likely to make a range of important household decisions (e.g. financial decisions, health care decisions, decisions on family visits) on their own, rather than in conjunction with their spouse (S6 Table in S1 File). Our use of male/female household heads therefore seems to reflect a general power and decision-making inequality within a household, but perhaps not to the full extent of differences across male and female-headed households.

Our results highlight fine-grained aspects of inequality, but can they also contribute additional information to national level policy discussions? To test this we looked to see if our results simply reproduce existing efforts to measure national level poverty and development indicators. We tested the results of our work against the United Nation’s (UN) Human Development Index, the UN Gender Inequality Index, and the UNDP’s Multidimensional Poverty Index. We also tested our metrics against a fundamental human health metric (infant mortality rate) and an established measure of economic inequality (Gini Coefficient). We found that country level averages of our gender inequality metrics show only very weak correlations with conventional, national-level poverty and development indicators, with absolute Pearson *r* values ranging from 0.03–0.38 (S7 Table in S1 File). This suggests that the results of our fine-scale analyses provide a novel perspective on inequality and well-being, even when aggregated to national levels.

## Discussion

Our results demonstrate the magnitude of the gap in land and asset ownership between male and female-headed households across 47 countries. Similar results have been found using more limited datasets at smaller spatial extents. For example, Doss et al. [24] used the World Bank's Living Standards and Measurement Surveys to show that for five African countries, rates of (sole) male ownership of land are between 1.29 and 5 times greater than for females. And in Latin America, Deere and Leon [25] found a large inequality in land ownership related to gender biases in inheritance practices, land markets and land distribution policies and programs. Despite the national scale results, our analysis shows the heterogeneity of these inequalities at scales that are appropriate for programs and interventions. Additionally, the heterogeneity supports the work of others to question the sweeping generalization that “female-headed households are the poorest of the poor” [26,27,28].

With a sample size of nearly 700,000 households, our results and high-resolution maps of multidimensional gender inequality are representative of a large part of the globe where important policy decisions regarding poverty alleviation suffer from data deficiencies, particularly at disaggregated sub-national, village, and household levels. Indeed, given the high degree of intra-country heterogeneity, our results suggest that focusing only on national averages may result in suboptimal or ineffective investments. Further, the fact that asset wealth inequality and land inequality are not strongly correlated in space suggests that targeting different areas across these domains will be necessary. The results we present here can be utilized by agencies to target gender and development programs, as well as by the research community to ground truth causal explanations for the patterns we describe.

We attempted to assemble the most inclusive dataset from USAID’s Demographic and Health Surveys, which is a program to provide data to decision makers on population, health and nutrition status across much of the developing world. However, it is important to note that the DHS data we used to develop our high-resolution maps of inequality are unavailable for parts of the developing world, including a lack of coverage in regions known to have substantial gender inequalities (e.g., the Middle East). Furthermore, we have purposefully refrained from any attempt to pin down specific causal explanations for the gender inequality patterns we have observed. One general mechanism underlying the patterns we have observed is that in many areas in developing countries, women lack the rights, knowledge or capital to secure their land and asset inheritance after being widowed, and such events can have long term impact on livelihood opportunities [29, 30]. As such, gender inequality in these regions is entrenched in the cultural, political and market systems that operate at household, community and national levels [25,31]. To understand such dynamics at the subnational or even village level will require detailed follow-up research that our mapping now allows.

One critique of our approach is the use of household headship as a proxy for gender disparities. This delineation is certainly imperfect and does not explicitly measure intra-household dynamics between males and females [23, 26, 31]. There is also the case that culturally defined ideas of “head” and “household” may affect how survey respondents answer questions [26]. There are two ways our use of headship may bias results. First, if on average, females in male-headed households jointly share in and own assets and land, our results represent an overestimate of the *total* gender inequality ([32], but see S6 Table in S1 File). Second, if on average, females in male-headed households have no rights or ownership to land and assets, our results represent an underestimate of the *total* gender inequality.

There is a long literature on the division of labor within a household and evidence that the burden of caring for the previous and future generations fall largely to females, as such the burden of the main within-household duties may inhibit the ability of women to share and own financial and capital assets [33]. Additionally, a closer look at the differences across female-headed households (e.g. age of household head) might be the more important indicator for understanding key correlates of poverty and inequality [28]. Further investigation is needed to understand the potential direction of bias in our results in particular locations, as well as how female-headship may create opportunities (eg. control of income) or impediments (lower levels of labor capital) in certain regions [28].

Our work is a snapshot in time for each country; further investigation is needed to better understand the temporal dynamics of inequality at comparably detailed resolutions. Such temporal dynamics are of critical concern since overarching global changes in access to markets, climatic conditions, and the availability of natural resources and ecosystem services may intensify disparities in income, assets, and power between men and women [34, 35]. Nevertheless, because DHS surveys are generally conducted at five-year intervals and ask consistent questions, our results can be readily updated over time and be used to track progress towards SDGs and other goals. Our spatial analysis of gender inequality can also serve as a starting point for studies on the drivers of inequality and their wider economic consequences. Once we understand both the patterns and the processes driving gender inequality, our ability to target interventions to propel the Sustainable Development Goals forward, and track the successes and failures spatially, will be dramatically improved.

## Supporting Information

S1 File Contains S1-S2 Figs and S1-S7 Tables. **S1 Fig:** Wealth ratios as a function of distance from coast in West Africa. **S2 Fig:** Relationship between altitude and wealth ratios in the Andes. **S1 Table:** National scale t-test results for wealth index score between male-headed households and female-headed households. **S2 Table:** National scale t-test results for land ownership between male-headed households and female-headed households. **S3 Table:** Inequality ratios (male-headed/female-headed) for asset wealth. **S4 Table:** Inequality ratios (male-headed/female-headed) for land. **S5 Table:** Differences in Household size numbers between male- and female-headed households when controlling for the lack of a male head in female-headed households. **S6 Table:** Statistical relationships between household head and household decision-making. **S7 Table:** National-level correlation across development, inequality indicators, and the wealth and land ratios.
(PDF)

## References

[1] UN. Open Working Group proposal for Sustainable Development Goals. Open Working Group of the General Assembly. 2015;(document A/68/970). Available: http://undocs.org/A/68/970.

[2] Smith LC, Khan F, Frankenberger TR, Wadud AKMA. Admissible Evidence in the Court of Development Evaluation? The Impact of CARE's SHOUHARDO Project on Child Stunting in Bangladesh. World Development. 2013;41:196–216. 10.1016/j.worlddev.2012.06.018 .

[3] Duflo E. Women Empowerment and Economic Development. Journal of Economic Literature. 2012;50(4):1051–79. 10.1257/jel.50.4.1051 .

[4] Chowdhury AMR, Bhuiya A, Chowdhury ME, Rasheed S, Hussain Z, Chen LC. Bangladesh: Innovation for Universal Health Coverage 1 The Bangladesh paradox: exceptional health achievement despite economic poverty. Lancet. 2013;382(9906):1734–45. 10.1016/s0140-6736(13)62148-0 . 24268002

[5] Giroux SC, Eloundou-Enyegue PM. Schooling Dividends from Fertility Transitions. Early Evidence for sub-Saharan Africa. Journal of Children and Poverty. 2013.

[6] Black RE, Allen LH, Bhutta ZA, Caulfield LE, de Onis M, Ezzati M, et al Maternal and child undernutrition 1—Maternal and child undernutrition: global and regional exposures and health consequences. Lancet. 2008;371(9608):243–60. 10.1016/s0140-6736(07)61690-0 . 18207566

[7] Abuya BA, Ciera J, Kimani-Murage E. Effect of mother's education on child's nutritional status in the slums of Nairobi. Bmc Pediatrics. 2012;12 8010.1186/1471-2431-12-80. . 10.1186/1471-2431-12-80 PMC3444953 22721431

[8] Juma SA. Men, women and natural resources in Kwale District, Kenya. Ambio. 1998;27(8):758–9. .

[9] Westermann O, Ashby J, Pretty J. Gender and social capital: The importance of gender differences for the maturity and effectiveness of natural resource management groups. World Development. 2005;33(11):1783–99. 10.1016/j.worlddev.2005.04.018 .

[10] Coleman EA, Mwangi E. Women's participation in forest management: A cross-country analysis. Global Environmental Change-Human and Policy Dimensions. 2013;23(1):193–205. 10.1016/j.gloenvcha.2012.10.005 .

[11] Kevane M. Gendered Production and Consumption in Rural Africa. Proceedings of the National Academy of Sciences. 2012;109(31):12350–5.

[12] USAID. Feed the Future: The US Government’s Global Hunger and Food Security Initiative 2012.

[13] DfID. Strategic Visions for Girls and Women London: Department for International Development, UK Government, 2011.

[14] WHO. Gender, Health and Malaria. WHO Department of Gender, Women and Health, 2007.

[15] UNEP. UNEP Gender Action Plan. 2006.

[16] Derose LF, Kravdal O. Educational reversals and first-birth timing in sub-Saharan Africa: A dynamic multilevel approach. Demography. 2007;44(1):59–77. 10.1353/dem.2007.0001 . 17461336

[17] Lloyd CB, Hewett P. Educational Inequalities in the Midst of Persistent Poverty. Journal of International Development. 2009;21(8):1137–51. 10.1012/jid.1650 .

[18] OECD. Gender Equality and the MDGs: What are the missing dimensions? 2010.

[19] WEF. Global Gender Gap Index. Geneva: World Economic Forum, 2013.

[20] ICF International. Demographic and Health Surveys. ICF International, Calverton, MD, USA: 2012.

[21] Jayne TS, Yamano T, Weber MT, Tschirley D, Rui BF, Chapoto A, et al Smallholder income and land distribution in Africa: implications for poverty reduction strategies. Food Policy. 2003;28(3):253–75. 10.1016/s0306-9192(03)00046-0 .

[22] Rutstein SO, Guillermo R. Guide to DHS Statistics: Demographic and Health Surveys. Calverton, MD: ORC Macro, 2006.

[23] Deere CD, Alvarado GE, Twyman J. Gender Inequality in Asset Ownership in Latin America: Female Owners vs Household Heads. Development and Change. 2012;43(2):505–30. 10.1111/j.1467-7660.2012.01764.x .

[24] Doss C, Kovarik C, Peterman A, Quisumbing A, van den Bold M. Gender inequalities in ownership and control of land in Africa: myth and reality. Agricultural Economics. 2015;46(3):403–34. 10.1111/agec.12171 .

[25] Deere CD, Leon M. The gender asset gap: Land in Latin America. World Development. 2003;31(6):925–47. 10.1016/s0305-750x(03)00046-9 .

[26] Buvinic M, Gupta GR. Female-headed households and female-maintained families: Are they worth targeting to reduce poverty in developing countries? Economic Development and Cultural Change. 1997;45(2):259–80. 10.1086/452273 .

[27] Chant S. Women-headed households: Poorest of the poor?—Perspectives from Mexico, Costa Rica and the Philippines. Ids Bulletin-Institute of Development Studies. 1997;28(3):26–48. .

[28] Chant S. The 'feminisation of poverty' and the 'feminisation' of anti-poverty programmes: Room for revision? Journal of Development Studies. 2008;44(2):165–97. 10.1080/00220380701789810 .

[29] Cooper E, Bird K. Inheritance: A Gendered and Intergenerational Dimension of Poverty. Development Policy Review. 2012;30(5):527–41. 10.1111/j.1467-7679.2012.00587.x .

[30] McPeak JG, Doss CR. Are household production decisions cooperative? Evidence on pastoral migration and milk sales from northern Kenya. American Journal of Agricultural Economics. 2006;88(3):525–41. 10.1111/j.1467-8276.2006.00877.x .

[31] Vijaya RM, Lahoti R, Swaminathan H. Moving from the Household to the Individual: Multidimensional Poverty Analysis. World Development. 2014;59:70–81. 10.1016/j.worlddev.2014.01.029 .

[32] Deere CD, Oduro AD, Swaminathan H, Doss C. Property rights and the gender distribution of wealth in Ecuador, Ghana and India. Journal of Economic Inequality. 2013;11(2):249–65. 10.1007/s10888-013-9241-z .

[33] Kabeer N. Gendered Poverty Traps: Inequality and Care in a Globalised World. European Journal of Development Research. 2011;23(4):527–30. 10.1057/ejdr.2011.29 .

[34] Weeratunge N, Snyder KA, Sze CP. Gleaner, fisher, trader, processor: understanding gendered employment in fisheries and aquaculture. Fish and Fisheries. 2010;11(4):405–20. 10.1111/j.1467-2979.2010.00368.x .

[35] Ketlhoilwe MJ. Improving resilience to protect women against adverse effects of climate change. Climate and Development. 2013;5(2):153–9. 10.1080/17565529.2013.789788 .

