# Supplementary material for: The Geography of Gender Inequality
Source: PLoS One. 2016 Mar 1;11(3):e0145778. doi: 10.1371/journal.pone.0145778 (PMC4773071; doi:10.1371/journal.pone.0145778)
Supplement: S1 File — S1 Fig: Wealth ratios as a function of distance from coast in West Africa. S2 Fig: Relationship between altitude and wealth ratios in the Andes. S1 Table: National scale t-test results for wealth index score between male-headed households and female-headed households. S2 Table: National scale t-test results for land ownership between male-headed households and female-headed households. S3 Table: Inequality ratios (male-headed/female-headed) for asset wealth. S4 Table: Inequality ratios (male-headed/female-headed) for land. S5 Table: Differences in Household size numbers between male- and female-headed households when controlling for the lack of a male head in female-headed households. S6 Table: Statistical relationships between household head and household decision-making. S7 Table: National-level correlation across development, inequality indicators, and the wealth and land ratios. (PDF) [file pone.0145778.s001.pdf]

Supporting Information

Demographic and Health Survey Analysis

The analysis regarding the wealth index and land ownership is based on responses from the Demographic and Health Surveys (1). Specific questions from the DHS survey were:

- HV271 in the household recode, which is a wealth index, based on principle components analysis across a given country (see ref 2).
- SH123/HV245 in the household recode which has some variant of this question "How many hectares of agricultural land do you own?"

The analysis regarding decision-making within the household is based on responses from the Demographic and Health Surveys (1, Table S6). Specific questions from the DHS survey were:

- V739 Who decides how to spend money?
- V743A Who has the final say on own health care?
- V743B Who has the final say on making large household purchases?
- V743D Who has the final say on visits to family or relatives?

S1 Fig. Wealth ratios as a function of distance from coast in West Africa. Belts of wealthier, female-headed households are located 800-1000 km from the coast, while wealthier male-headed households are found 400-600 km away; both inequality belts extend from Senegal through several intervening countries all the way to Cameroon (Fig. 2c).

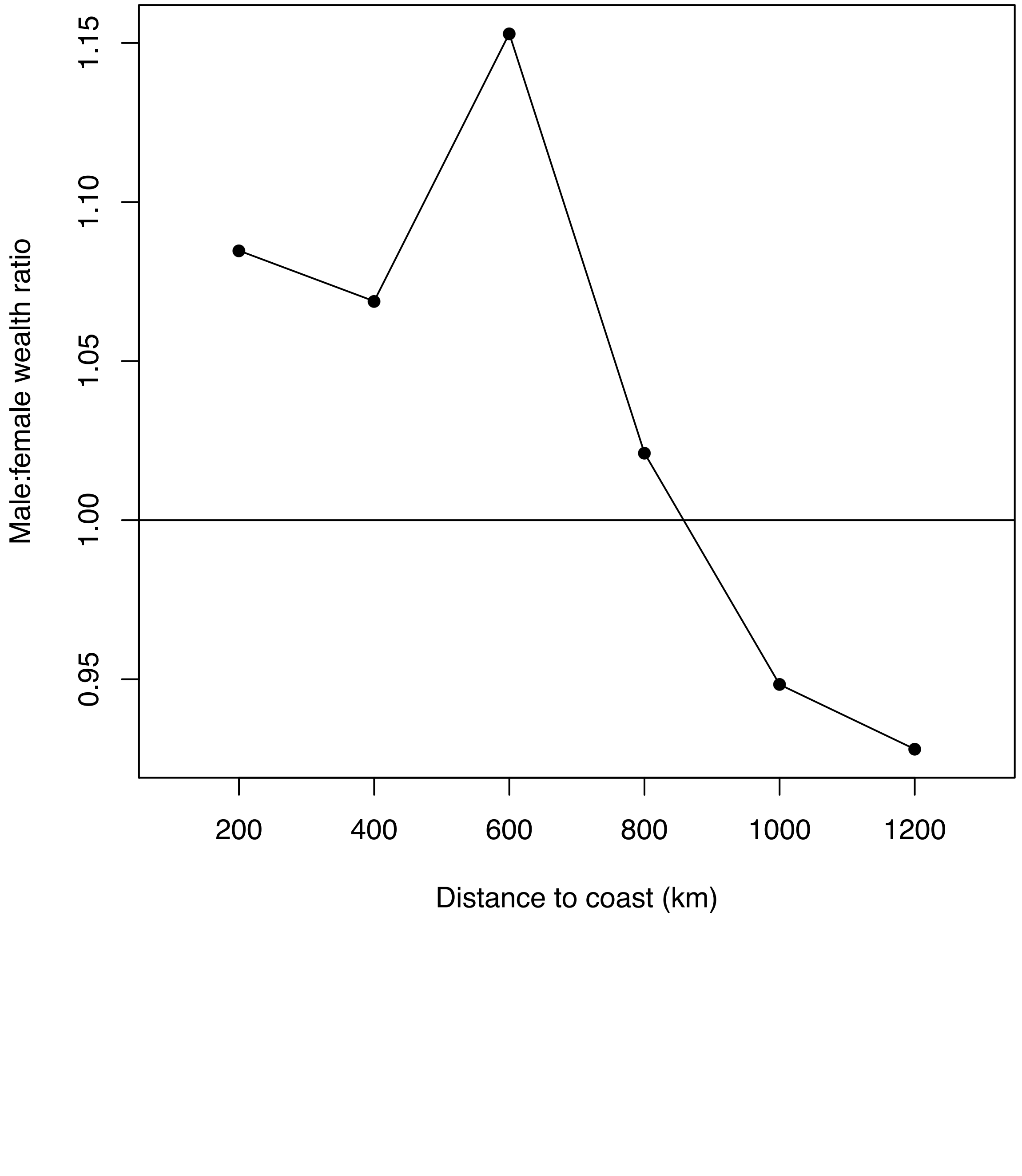

S2 Fig. Relationship between altitude and wealth ratios in the Andes

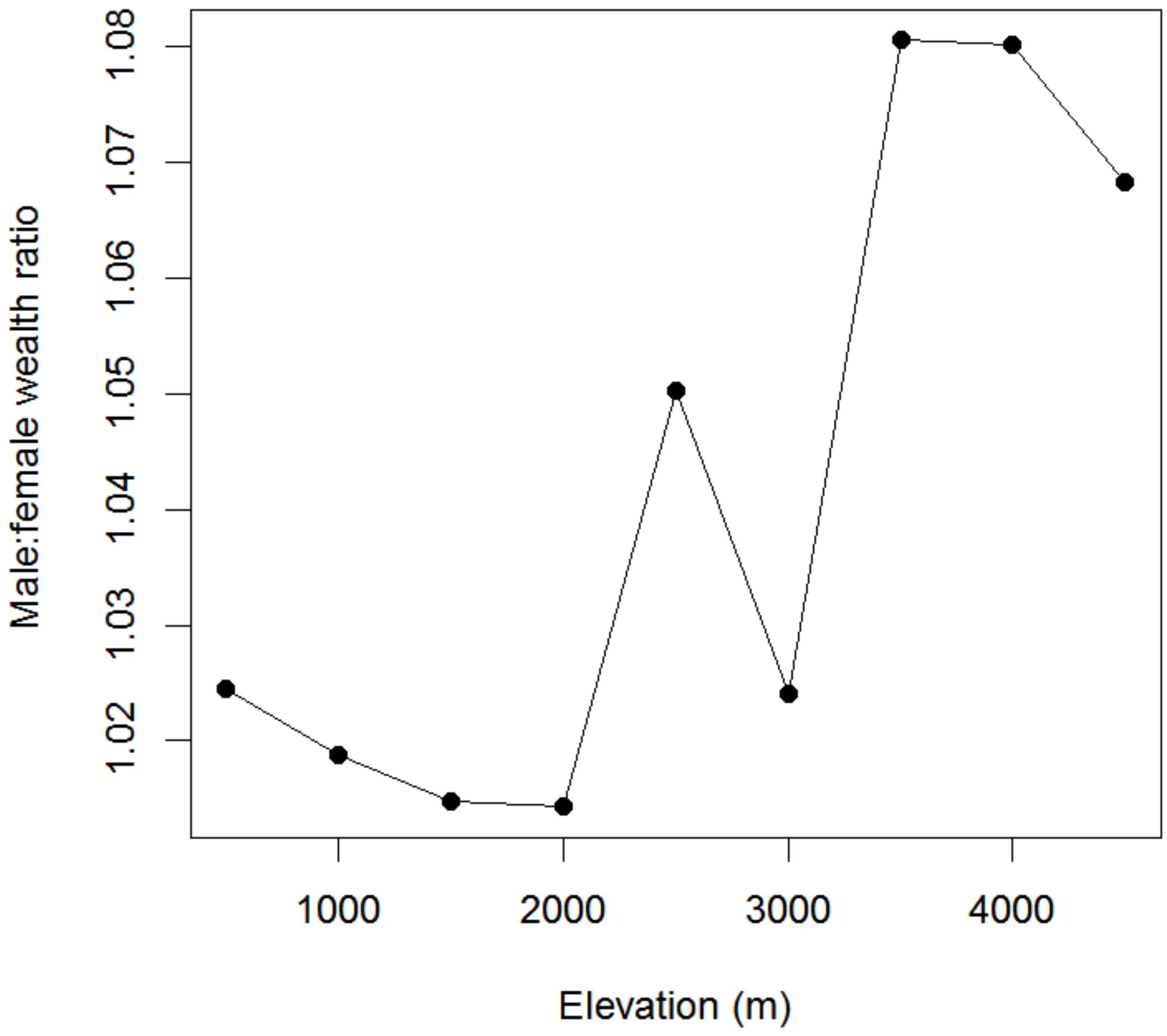

S1 Table. National scale t-test results for wealth index score between male-headed households and female-headed households.

Country	Survey Year	Average Wealth (male)	Average Wealth (Female)	df	t.value	p.value	Wealth Ratio (male/ female)	Significant
Angola	2011	1583531.624	1451801.401	2821	1.6626	0.0971	1.0907	-
Albania	2008	239405.1334	229360.079	1860	1.5054	0.1327	1.0438	-
Bangladesh	2011	154407.8176	146810.4592	2374	1.4992	0.1341	1.0517	-
Burkina Faso	2010	94217.28972	97052.80425	1782	-0.5276	0.5979	0.9708	-
Benin	2001	1.190285855	1.157565171	1897	0.3787	0.7051	1.0283	-
Bolivia	2008	210794.5048	206518.5645	7959	1.0498	0.2939	1.0207	-
Burundi	2010	64846.55644	50240.65317	5285	2.3161	0.0208	1.2907	*
Congo, Democratic Republic	2007	110527.4963	98859.421	3064	1.5398	0.1242	1.1180	-
Central African Republic	1994	0.932627685	0.749973986	2153	2.2807	0.0230	1.2435	*
Cote d'Ivoire	2012	130052.9191	109616.872	2746	3.2783	0.0011	1.1864	*
Cameroon	2011	193144.4783	182779.0551	6729	2.0236	0.0432	1.0567	*
Colombia	2010	317014.9594	320061.335	39166	-1.5743	0.1154	0.9905	-
Dominican Republic	2007	226489.7104	228626.0487	23950	-0.6474	0.5174	0.9907	-
Egypt	2008	329576.0437	306234.7604	3240	6.1778	0.0000	1.0762	*
Ethiopia	2010	212227.5411	207884.9562	8241	0.8578	0.3912	1.0209	-
Ghana	2008	236801.8041	222243.9308	8533	2.3875	0.0172	1.0655	*
Guinea	2005	103084.719	108872.4278	1538	-0.6644	0.5067	0.9468	-

Guyana	2009	243745.3896	244285.7199	4079	-0.0646	0.9485	0.9978	-
Haiti	2006	163447.3213	158927.7017	9168	0.6560	0.5121	1.0284	-
Indonesia	2002	202337.8737	185958.6691	4636	4.7065	0.0000	1.0881	*
Jordan	2007	385397.9682	345884.0618	1869	14.2154	0.0000	1.1142	*
Kenya	2009	217050.9737	213314.0664	6480	0.5551	0.5790	1.0175	-
Cambodia	2010	151905.8965	134027.4983	6999	3.6237	0.0003	1.1334	*
Liberia	2011	146966.6594	134689.9912	2756	1.2034	0.2298	1.0911	-
Lesotho	2009	159065.1675	142470.0624	7503	2.8463	0.0045	1.1165	*
Morocco	2004	219229.7181	214098.8203	2863	0.8481	0.3966	1.0240	-
Moldova	2005	209470.0898	195996.3056	7801	2.0766	0.0382	1.0687	*
Madagascar	2008	117851.5854	107739.5847	7031	1.9978	0.0460	1.0939	*
Mali	2006	107002.6938	116878.5894	2106	-1.5184	0.1293	0.9155	-
Malawi	2010	127832.7987	108346.9969	14731	4.6688	0.0000	1.1798	*
Mozambique	2011	118874.1772	109720.9144	11598	1.6997	0.0894	1.0834	-
Nigeria	2008	148338.6771	142693.6749	10050	1.2743	0.2028	1.0396	-
Niger	1998	0.930091175	0.861419146	1245	0.8282	0.4080	1.0797	-
Namibia	2007	133072.9687	127853.107	8632	0.6814	0.4959	1.0408	-
Nepal	2011	161579.6056	154358.0386	5650	1.0143	0.3109	1.0468	-
Peru	2004	176828.6704	169615.2339	15076	2.5735	0.0101	1.0425	*
Philippines	2008	224039.0274	230418.6778	2939	-1.4907	0.1363	0.9723	-
Rwanda	2010	170227.9717	154297.2683	10106	3.0758	0.0022	1.1032	*
Sierra Leone	2008	138976.381	134828.081	2588	0.6433	0.520	1.0308	-

		4	6			2		
Senegal	2011	170911.0135	185079.6718	2793	-2.1861	0.0292	0.9234	*
Swaziland	2006	214879.4644	207679.0932	4811	1.4068	0.1598	1.0347	-
Timor Leste	2009	112453.1646	97075.20329	1921	3.2723	0.0011	1.1584	*
Togo	98	1.63244648	1.544092205	2950	1.1636	0.2451	1.0572	-
Tanzania	2010	117385.412	97107.65866	4253	3.6499	0.0003	1.2088	*
Uganda	2011	147257.0605	137553.3673	5312	1.5591	0.1194	1.0705	-
Zambia	2007	113698.1676	110810.5217	3277	0.3875	0.6985	1.0261	-
Zimbabwe	2005	136689.7099	134548.3508	7681	0.3268	0.7439	1.0159	-

S2 Table. National scale t-test results for land ownership between male-headed households and female-headed households. Land data was not available for 17 of our 47 Demographic and Health Survey countries.

Country	Survey Year	Average Land (male)	Average Land (Female)	t.value	p.value	Land Ratio (male/female)	Significant
Albania	2008	1.3688	1.2398	0.3303	0.7414	1.1040	-
Bangladesh	2011	0.3720	0.4096	-0.6196	0.5358	0.9082	-
Burkina Faso	2010	3.2899	2.1198	9.4787	<0.0001	1.5520	*
Burundi	2010	1.6088	1.2398	2.4894	0.0130	1.2977	*
Cote d'Ivoire	2012	9.0133	6.5655	3.2179	0.0014	1.3728	*
Cameroon	2011	6.5717	4.9472	2.1791	0.0296	1.3284	*
Dominican Republic	2007	4.6539	3.9827	1.2520	0.2108	1.1685	-
Ethiopia	2010	5.3181	4.8059	0.8635	0.3881	1.1066	-
Ghana	2008	3.0424	1.8435	5.5207	<0.0001	1.6503	*
Guyana	2009	9.3523	7.5749	0.9412	0.3478	1.2346	-
Haiti	2006	4.5495	4.3366	0.2296	0.8185	1.0491	-
Kenya	2009	2.8075	3.0754	-0.7333	0.4638	0.9129	-
Cambodia	2010	2.0930	1.3288	3.9370	0.0001	1.5751	*
Lesotho	2009	5.0644	4.8388	0.7217	0.4707	1.0466	-
Madagascar	2008	2.0866	1.3796	3.8066	0.0001	1.5124	*
Malawi	2010	5.8813	4.9253	5.2386	0.0000	1.1941	*
Mozambique	2011	2.7970	2.2891	2.5171	0.0120	1.2218	*
Nigeria	2008	20.1755	20.6609	-0.4377	0.6618	0.9765	-
Namibia	2007	22.1196	21.7874	0.0746	0.9406	1.0152	-
Nepal	2011	0.7299	0.4813	5.2220	<0.0001	1.5166	*
Peru	2004	5.3501	4.7616	1.4565	0.1454	1.1236	-
Rwanda	2010	0.9510	0.7606	3.8327	0.0001	1.2504	*
Sierra Leone	2008	13.3022	13.1659	0.0700	0.9443	1.0104	-
Senegal	2011	2.3801	3.4958	-3.8573	0.0001	0.6809	*
Swaziland	2006	77.5963	76.2903	0.6076	0.5437	1.0171	-
Timor Leste	2009	3.6081	4.5446	-1.0772	0.2820	0.7939	-
Tanzania	2010	1.4844	0.9863	5.2143	<0.0001	1.5051	*
Uganda	2011	1.9740	1.6420	1.7242	0.0851	1.2022	-
Zambia	2007	4.8692	2.7646	4.2955	<0.0001	1.7613	*
Zimbabwe	2005	4.6193	5.6360	-1.8487	0.0650	0.8196	-

S3 Table. Inequality ratios (male-headed/female-headed) for asset wealth, coefficients (with p-values in brackets) on gender and urban/rural interaction tests, number of villages sampled, and number of households sampled, for 47 Demographic and Health Survey countries.

Country	Survey year	Wealth ratio	Female-headed (*p*-value)	Rural (*p*-value)	Female-headed x rural (*p*-value)	No. villages	No. households
Angola	2011	1.89	-30256 (0.377)	-995548 (<0.0001)	-139099 (0.002)	238	8030
Albania	2008	1.96	-11502 (< 0.0001)	-159350 (<0.0001)	1694 (0.655)	389	7999
Bangladesh	2011	1.96	2040 (0.561)	-110645 (<0.0001)	1305 (0.763)	598	17141
Burkina Faso	2010	3.58	2577 (0.429)	-135546 (<0.0001)	-2582 (0.545)	565	14424
Benin	2001	3.09	0.085 (0.044)	-1.209 (<0.0001)	-0.008 (0.878)	247	5769
Bolivia	2008	2.35	-5616 (< 0.0001)	-159338 (<0.0001)	14426 (<0.0001)	1000	19564
Burundi	2010	7.06	-44199 (< 0.0001)	-178862 (<0.0001)	36483 (<0.0001)	374	8596
Congo, Democratic Republic	2007	3.71	-16675 (< 0.0001)	-139175 (<0.0001)	9029 (0.019)	300	8886
Central African Republic	1994	3.66	-0.257 (< 0.0001)	-1.0941 (<0.0001)	0.178 (0.002)	231	5551
Cote d'Ivoire	2012	3.04	-24324 (< 0.0001)	-137428 (<0.0001)	201 (0.951)	336	9686
Cameroon	2011	2.16	-13514 (< 0.0001)	-142034 (<0.0001)	10947 (<0.0001)	576	14214
Colombia	2010	1.76	-603 (0.422)	-161057 (<0.0001)	18740 (<0.0001)	4987	51447
Dominican Republic	2007	1.52	9674 (< 0.0001)	-95883 (<0.0001)	12744 (<0.0001)	1232	32431
Egypt	2008	1.41	-24318 (< 0.0001)	-112327 (<0.0001)	-1920 (0.588)	1267	18968
Ethiopia	2010	2.06	-4013 (0.012)	-178059 (<0.0001)	-21.391 (0.991)	538	16702
Ghana	2008	1.74	-23724 (< 0.0001)	-142648 (<0.0001)	36745 (<0.0001)	399	11778
Guinea	2005	4.84	-12640 (0.001)	-173220 (<0.0001)	15770 (0.001)	295	6282
Guyana	2009	1.34	189 (0.968)	-88732	17260 (0.002)	210	5632

				(<0.0001)			
Haiti	2006	2.25	-5.837 (0.998)	-134875 (<0.0001)	6091 (0.051)	316	9998
Indonesia	2002	1.81	-22508 (< 0.0001)	-121393 (<0.0001)	5769 (0.048)	1392	33088
Jordan	2007	1.14	-38712 (< 0.0001)	-48489 (<0.0001)	-5019 (0.368)	1860	14564
Kenya	2009	2.02	-11524 (< 0.0001)	-159863 (<0.0001)	-972 (0.762)	379	9057
Cambodia	2010	2.34	-15268 (< 0.0001)	-140929 (<0.0001)	1192 (0.704)	596	15667
Liberia	2011	2.58	-22002 (< 0.0001)	-134477 (<0.0001)	7981 (0.108)	150	4162
Lesotho	2009	2.24	-26370 (< 0.0001)	-173965 (<0.0001)	17875 (<0.0001)	388	9391
Morocco	2004	2.21	-12526 (< 0.0001)	-158289 (<0.0001)	10665 (0.001)	480	11513
Moldova	2005	2.21	-8444 (< 0.0001)	-147521 (<0.0001)	2471 (0.386)	400	11095
Madagascar	2008	3.07	-29271 (< 0.0001)	-167279 (<0.0001)	24795 (<0.0001)	583	17857
Mali	2006	3.28	-14112 (< 0.0001)	-137898 (<0.0001)	29739 (<0.0001)	407	12998
Malawi	2010	2.82	-29213 (< 0.0001)	-167034 (<0.0001)	7689 (0.046)	849	24825
Mozambique	2011	3.45	-11152 (< 0.0001)	-147323 (<0.0001)	15552 (<0.0001)	603	13919
Nigeria	2008	2.07	-14475 (< 0.0001)	-158473 (<0.0001)	30615 (<0.0001)	783	34070
Niger	1998	5.87	-0.523 (< 0.0001)	-1.7366 (<0.0001)	0.515 (<0.0001)	268	5928
Namibia	2007	2.31	-10475 (< 0.0001)	-119246 (<0.0001)	-14913 (<0.0001)	254	9200
Nepal	2011	1.96	-9267 (0.007)	-117066 (<0.0001)	5805 (0.153)	289	10826
Peru	2004	2.42	-3119 (0.001)	-139300 (<0.0001)	-4516 (0.005)	1970	46073
Philippines	2008	1.53	13996 (< 0.0001)	-91831 (<0.0001)	2863 (0.507)	792	12469
Rwanda	2010	2.11	-52050 (< 0.0001)	-166130 (<0.0001)	37935 (<0.0001)	491	12540
Sierra Leone	2008	2.45	-14359 (< 0.0001)	-141021 (<0.0001)	11911 (0.004)	317	7284

Senegal	2011	2.00	6916 (0.022)	-134890 (<0.0001)	36884 (<0.0001)	364	7902
Swaziland	2006	1.72	-18027 (< 0.0001)	-123590 (<0.0001)	6933 (0.15)	486	4843
Timor Leste	2009	2.17	-36626 (< 0.0001)	-108594 (<0.0001)	27910 (<0.0001)	442	11463
Togo	1998	2.52	-0.221 (< 0.0001)	-1.5327 (<0.0001)	0.412 (<0.0001)	288	7517
Tanzania	2010	3.02	-36270 (< 0.0001)	-156632 (<0.0001)	20067 (<0.0001)	475	9623
Uganda	2011	2.49	-17104 (< 0.0001)	-152329 (<0.0001)	9233 (0.012)	403	9033
Zambia	2007	3.72	-11361 (< 0.0001)	-157210 (<0.0001)	3488 (0.341)	313	7164
Zimbabwe	2005	3.20	3868 (0.071)	-175919 (<0.0001)	-10038 (<0.0001)	389	9285

S4 Table. Inequality ratios (male-headed/female-headed) for land, coefficients (with p-values in brackets) on gender and urban/rural interaction tests, number of villages sampled, and number of households sampled, for 47 Demographic and Health Survey countries.

Country	Survey year	Land ratio	Female-headed (*p*-value)	Rural (*p*-value)	Female-headed x rural (*p*-value)	No. villages	No. house-holds
Angola	2011	-	-	-	-	238	8030
Albania	2008	1.60	-1.036 (0.0818)	-0.765 (0.0005)	0.783 (0.2208)	389	7999
Bangladesh	2011	4.60	0.118 (0.0834)	-0.575 (<0.0001)	-0.064 (0.4304)	598	17141
Burkina Faso	2010	0.84	-1.136 (0.0005)	1.061 (0.0112)	-0.508 (0.1657)	565	14424
Benin	2001	-	-	-	-	247	5769
Bolivia	2008	-	-	-	-	1000	19564
Burundi	2010	2.06	-1.035 (0.0103)	-1.427 (<0.0001)	0.677 (0.109)	374	8596
Congo, Democratic Republic	2007	-	-	-	-	300	8886
Central African Republic	1994	-	-	-	-	231	5551
Cote d'Ivoire	2012	1.13	-3.472 (0.0008)	-1.344 (0.0062)	0.984 (0.404)	336	9686
Cameroon	2011	0.88	-2.124 (0.0013)	0.367 (0.3673)	0.96 (0.2261)	576	14214
Colombia	2010	-	-	-	-	4987	51447
Dominican Republic	2007	1.89	0.563 (0.3789)	-2.458 (<0.0001)	-1.956 (0.015)	1232	32431
Egypt	2008	-	-	-	-	1267	18968
Ethiopia	2010	1.16	1.117 (0.1607)	-0.346 (0.411)	-1.285 (0.1206)	538	16702
Ghana	2008	1.20	-0.625 (0.0523)	-0.435 (0.0282)	-0.467 (0.2057)	399	11778
Guinea	2005	-	-	-	-	295	6282
Guyana	2009	1.70	-9.3 (0.0362)	-7.195 (0.0001)	8.12 (0.0834)	210	5632
Haiti	2006	1.12	-0.564 (0.6297)	-0.715 (0.4023)	0.456 (0.7404)	316	9998
Indonesia	2002	-	-	-	-	1392	33088
Jordan	2007	-	-	-	-	1860	14564
Kenya	2009	1.99	0.733 (0.0771)	-1.956 (<0.0001)	-0.743 (0.0962)	379	9057

Cambodia	2010	1.70	-1.301 (<0.0001)	-1.191 (<0.0001)	0.547 (0.04)	596	15667
Liberia	2011	-	-	-	-	150	4162
Lesotho	2009	1.09	-1.483 (0.0448)	-2.193 (0.0422)	1.314 (0.0849)	388	9391
Morocco	2004	-	-	-	-	480	11513
Moldova	2005	-	-	-	-	400	11095
Madagascar	2008	1.54	-0.823 (0.0164)	-0.972 (<0.0001)	0.187 (0.6097)	583	17857
Mali	2006	-	-	-	-	407	12998
Malawi	2010	0.80	-0.362 (0.4115)	1.532 (<0.0001)	-1.154 (0.0109)	849	24825
Mozambique	2011	1.25	-0.348 (0.1931)	-0.59 (0.0011)	-0.028 (0.9236)	603	13919
Nigeria	2008	1.04	-2.003 (0.1821)	-1.476 (0.4332)	0.593 (0.7178)	783	34070
Niger	1998	-	-	-	-	268	5928
Namibia	2007	1.98	6.755 (0.313)	-10.021 (0.0041)	-21.935 (0.0018)	254	9200
Nepal	2011	1.64	-0.24 (0.0006)	-0.331 (<0.0001)	0.04 (0.6107)	289	10826
Peru	2004	1.67	-1.747 (0.0011)	-3.055 (<0.0001)	-0.065 (0.9152)	1970	46073
Phillipines	2008	-	-	-	-	792	12469
Rwanda	2010	1.45	-0.69 (<0.0001)	-0.537 (<0.0001)	-	491	12540
Sierra Leone	2008	0.82	-2.272 (0.3646)	2.773 (0.0302)	-1.298 (0.6425)	317	7284
Senegal	2011	1.62	1.864 (<0.0001)	-1.079 (<0.0001)	-1.001 (0.0116)	364	7902
Swaziland	2006	0.34	10.311 (0.0009)	60.919 (<0.0001)	-9.864 (0.0026)	486	4843
Timor Leste	2009	1.64	0.957 (0.4268)	-2.034 (<0.0001)	-1.06 (0.4298)	442	11463
Togo	1998	-	-	-	-	288	7517
Tanzania	2010	0.49	0.028 (0.8657)	0.93 (<0.0001)	-0.726 (0.0001)	475	9623
Uganda	2011	1.48	-0.457 (0.0726)	-0.792 (<0.0001)	0.215 (0.4401)	403	9033
Zambia	2007	1.13	-2.403 (0.0081)	-0.695 (0.1592)	0.807 (0.4192)	313	7164
Zimbabwe	2005	1.62	1.609 (0.0453)	-2.31 (<0.0001)	-1.218 (0.1492)	389	9285

S5 Table. Differences in Household size numbers between male- and female-headed households when controlling for the lack of a male head in female-headed households. Only in 2 countries do male-headed households have, on average, more than one more person than female-headed households. Across the dataset, male-headed households have on average 0.04 more people in the household.

Country	Survey Year	Household Size (male)	Household Size (female)	Difference - 1
Angola	2011	5.21	4.51	-0.30
Albania	2008	4.08	2.91	0.17
Bangladesh	2011	5.02	3.79	0.24
Burkina Faso	2010	5.87	4.16	0.71
Benin	2001	5.60	4.02	0.58
Bolivia	2008	4.23	3.14	0.09
Burundi	2010	5.29	3.94	0.35
Congo, Democratic Republic	2007	5.67	4.54	0.13
Central African Republic	94	5.34	3.99	0.34
Cote d'Ivoire	2012	5.44	4.59	-0.15
Cameroon	2011	5.42	4.22	0.20
Colombia	2010	4.12	3.69	-0.57
Dominican Republic	2007	3.89	3.66	-0.77
Egypt	2008	5.05	3.56	0.50
Ethiopia	2010	5.04	3.69	0.35
Ghana	2008	4.24	3.32	-0.08
Guinea	2005	6.40	4.50	0.89
Guyana	2009	4.20	3.75	-0.56
Haiti	2006	4.84	4.60	-0.76
Indonesia	2002	4.65	3.40	0.25
Jordan	2007	5.86	4.02	0.83
Kenya	2009	4.43	3.91	-0.48
Cambodia	2010	5.13	4.25	-0.11
Liberia	2011	4.76	4.50	-0.73
Lesotho	2009	4.95	4.38	-0.43
Morocco	2004	5.83	4.19	0.64
Moldova	2005	3.20	2.31	-0.11
Madagascar	2008	5.10	3.85	0.24
Mali	2006	5.88	4.18	0.70

Malawi	2010	5.04	4.14	-0.10
Mozambique	2011	4.82	3.99	-0.17
Nigeria	2008	4.91	3.22	0.69
Niger	98	6.51	4.17	1.34
Namibia	2007	4.50	4.81	-1.31
Nepal	2011	4.92	3.76	0.16
Peru	2004	4.28	3.26	0.01
Philipines	2008	5.04	4.07	-0.03
Rwanda	2010	4.84	3.83	0.01
Sierra Leone	2008	5.83	5.55	-0.72
Senegal	2011	10.20	8.28	0.92
Swaziland	2006	4.28	4.92	-1.64
Timor Leste	2009	6.15	4.27	0.88
Togo	98	6.33	4.24	1.09
Tanzania	2010	5.50	4.41	0.10
Uganda	2011	5.17	4.55	-0.37
Zambia	2007	5.25	4.14	0.11
Zimbabwe	2005	4.69	4.44	-0.75

Average Difference in Household Size **0.04**

S6 Table. Statistical relationships between household head and household decision-making. Coefficients (standard errors) from logistic regression models of household head on decision-making for finances (column 2), health care (column 3), large household purchases (column 4) and visits by family relatives (column 5). In each case, the baseline reference category is "respondent decides for themselves". Respondents are typically the oldest female in the household. Negative coefficients indicate that husbands in male-headed households would likely make such decisions alone as compared to by women themselves. *** - $p < 0.001$; ** - $p < 0.01$; * - $p < 0.05$; . - $p < 0.10$.

	**Financial decisions**	**Health care decisions**	**Large household purchases**	**Visits by family relatives**
**Country**	Husband alone	Husband alone	Husband alone	Husband alone
Albania	-0.991 (0.445)*	-0.928 (0.169)***	-1.006 (0.172)***	-1.01 (0.160)***
Angola	n/a	n/a	n/a	n/a
Bangladesh	-2.05 (0.515)***	-2.54 (0.097)***	-2.55 (0.096)***	-2.35 (0.092)***
Burkina Faso	-0.484 (0.262) .	-1.67 (0.090)***	-2.23 (0.110)***	-0.706 (0.081)***
Benin	-1.86 (0.342)***	-1.62 (0.086)***	-1.93 (0.090)***	-2.22 (0.103)***
Bolivia	-1.30 (0.329)***	-0.859 (0.131)***	-0.765 (0.106)***	-0.620 (0.129)***
Burundi	-1.20 (0.503)*	-1.180 (0.136)***	-1.21 (0.134)***	-1.25 (0.149)***
Central Afr. Rep.	-1.65 (0.254)***	n/a	n/a	n/a
Cote d'Ivoire	-0.239 (0.159)	-1.09 (0.110)***	-0.947 (0.114)***	-0.954 (0.101)***
Cambodia	-0.142 (0.275)	-0.363 (0.098)***	-0.334 (0.130)*	-0.615 (0.138)***
Cameroon	-0.873 (0.181)***	-1.06 (0.070)***	-1.24 (0.071)***	-1.11 (0.069)***
Colombia	-0.656 (0.160)***	-0.958 (0.291)**	-1.60 (0.119)***	-1.54 (0.082)***
Dem. Rep. Congo	-1.96 (0.128)***	-1.89 (0.069)***	-2.19 (0.073)***	-2.09 (0.072)***
Dominican Republic	-0.585 (0.160)***	-0.495 (0.067)***	-0.866 (0.061)***	-0.455 (0.066)***
Egypt	-0.829 (0.738)	-0.621 (0.136)***	-1.39 (0.128)***	-1.26 (0.133)***
Ethiopia	-0.869 (0.183)***	-0.793 (0.077)***	-1.08 (0.090)***	-0.694 (0.075)***
Ghana	-0.688 (0.213)**	-0.609 (0.114)***	-0.990 (0.113)***	-0.688 (0.138)***
Guinea	-0.889 (0.248)***	-1.51 (0.090)***	-1.70 (0.095)***	-1.58 (0.097)***

Guyana	-0.679 (0.430)	-0.367 (0.176)*	-0.344 (0.149)*	-0.398 (0.177)*
Haiti	-0.184 (0.198)	-0.613 (0.069)***	-0.644 (0.077)***	-0.814 (0.099)***
Indonesia	-1.29 (0.388)***	-1.14 (0.095)***	-2.20 (0.086)***	-2.06 (0.103)***
Jordan	-15.4 (931)	-0.270 (0.228)	-0.917 (0.189)***	-0.797 (0.201)***
Kenya	-1.00 (0.226)***	-0.259 (0.085)**	-0.549 (0.098)***	-0.564 (0.091)***
Lesotho	-1.081 (0.412)**	-0.466 (0.130)***	-0.658 (0.141)***	-0.082 (0.105)
Liberia	n/a	n/a	n/a	n/a
Madagascar	n/a	n/a	n/a	n/a
Mali	-2.88 (0.710)***	-1.88 (0.071)***	-1.81 (0.072)***	-1.37 (0.071)***
Malawi	n/a	n/a	n/a	n/a
Mozambique	-0.655 (0.172)***	-0.986 (0.068) ***	-0.740 (0.070)***	-0.742 (0.073)***
Morocco	-1.35 (0.285)***	-3.14 (0.112)***	-3.46 (0.113)***	-3.17 (0.115)***
Moldova	-1.22 (0.365)***	-0.285 (0.232)	-0.390 (0.188)*	-0.448 (0.230) .
Nigeria	n/a	n/a	n/a	n/a
Niger	-1.71 (0.509)***	n/a	n/a	n/a
Namibia	-0.744 (0.197)***	-0.319 (0.105)**	-0.353 (0.102)***	-0.470 (0.105)***
Nepal	-1.42 (0.284) ***	-2.07 (0.075)***	-2.42 (0.087)***	-2.33 (0.088)***
Peru	-1.32 (0.174)***	-1.88 (0.520)***	-2.11 (0.251)***	-1.84 (0.120)***
Phillipines	-0.839 (0.318)**	-0.578 (0.189)**	-0.489 (0.133)***	-0.378 (0.160)*
Rwanda	-2.06 (0.182)***	-2.01 (0.118)***	-2.38 (0.121)***	-1.86 (0.130)***
Senegal	-0.175 (0.142)	-0.712 (0.074)***	-0.962 (0.087)***	-0.711 (0.072)***
Sierra Leone	-0.567 (0.194)**	-0.572 (0.112)***	-0.561 (0.116)***	-0.417 (0.112)***
Swaziland	-0.143 (0.311)	-0.191 (0.111) .	-0.118 (0.125)	-0.461 (0.117)***
Timor Leste	0.066 (0.654)	-0.327 (0.190) .	-0.299 (0.185)	0.139 (0.221)
Togo	-1.73 (0.363)***	n/a	n/a	n/a
Uganda	n/a	n/a	n/a	n/a
Zambia	-0.949 (0.277)***	-0.403 (0.127)**	-0.275 (0.153) .	-0.504 (0.132)***
Zimbabwe	-0.613 (0.180) ***	-0.626 (0.093)***	-0.418 (0.100)***	-0.517 (0.104)***

S7 Table. National-level correlation across development, inequality indicators, and the wealth and land ratios.

	Wealth Ratio	Land Ratio	Human Development Index (2013)	Gender Inequality Index (2010)	Gini Coefficient (2012)	Infant Mortality Rate (2012)	Multi-Dimensional Poverty Index (2012)
**Wealth Ratio**	1	-	-	-	-	-	-
**Land Ratio**	0.341	1	-	-	-	-	-
**Human Development Index (2013)**	-0.381	-0.098	1	-	-	-	-
**Gender Inequality Index (2010)**	-0.145	0.031	-0.636	1	-	-	-
**Gini Coefficient (2012)**	-0.224	-0.057	0.084	0.133	1	-	-
**Infant Mortality Rate (2012)**	0.240	0.038	-0.816	0.672	0.054	1	-
**Multi-Dimensional Poverty Index (2012)**	0.317	-0.040	-0.880	0.474	-0.171	0.740	1

Sources

1. Human Development Index: Inequality-adjusted HDI value: Calculated as the geometric mean of the values in Columns 5, 7 and 9 using the methodology in Technical note 2.

2. Gender Inequality Index, value: HDRO calculations based on UN Maternal Mortality Estimation Group (MMEIG) WHO, UNICEF, UNFPA and the World Bank (2012), UNDESA (2011), IPU (2012), Barro and Lee (2010), UNESCO Institute for Statistics (2012) and ILO (2012).

3. Income Gini coefficient: World Bank (2012).

4. Under-five Infant Mortality Rate: Inter-agency Group for Child Mortality Estimation (UNICEF, WHO, UN Population Division and World Bank) 2012. Accessed March 2012. per 1000 live births.

5. Multidimensional Poverty Index: Calculated from various household surveys,

including ICF Macro Demographic and Health Surveys, United Nations Children's Fund Multiple Indicator Cluster Surveys and World Health Organization World Health Surveys conducted between 2000 and 2010.

Accessed: 10/9/2013,3:13 PM from: http://hdr.undp.org

References

1. ICF International, “Demographic and Health Surveys.”.MEASURE DHS - http://www.dhsprogram.com/Data/, ICF International, Calverton, MD, USA (2012)

2. S. O. Rutstein, K. Johnson, “The DHS Wealth Index. DHS Comparative Reports No. 6.” (ORC Macro, Calverton, MD, 2004).
